# omicsGAT: Graph Attention Network for Cancer Subtype Analyses

**DOI:** 10.3390/ijms231810220

**Published:** 2022-09-06

**Authors:** Sudipto Baul, Khandakar Tanvir Ahmed, Joseph Filipek, Wei Zhang

**Affiliations:** 1 Department of Computer Science, University of Central Florida, Orlando, FL 32816, USA; 2 Genomics and Bioinformatics Cluster, University of Central Florida, Orlando, FL 32816, USA

**Keywords:** graph attention network, single-cell RNA-seq, patient stratification, cancer outcome prediction

## Abstract

The use of high-throughput omics technologies is becoming increasingly popular in all facets of biomedical science. The mRNA sequencing (RNA-seq) method reports quantitative measures of more than tens of thousands of biological features. It provides a more comprehensive molecular perspective of studied cancer mechanisms compared to traditional approaches. Graph-based learning models have been proposed to learn important hidden representations from gene expression data and network structure to improve cancer outcome prediction, patient stratification, and cell clustering. However, these graph-based methods cannot rank the importance of the different neighbors for a particular sample in the downstream cancer subtype analyses. In this study, we introduce omicsGAT, a graph attention network (GAT) model to integrate graph-based learning with an attention mechanism for RNA-seq data analysis. The multi-head attention mechanism in omicsGAT can more effectively secure information of a particular sample by assigning different attention coefficients to its neighbors. Comprehensive experiments on The Cancer Genome Atlas (TCGA) breast cancer and bladder cancer bulk RNA-seq data and two single-cell RNA-seq datasets validate that (1) the proposed model can effectively integrate neighborhood information of a sample and learn an embedding vector to improve disease phenotype prediction, cancer patient stratification, and cell clustering of the sample and (2) the attention matrix generated from the multi-head attention coefficients provides more useful information compared to the sample correlation-based adjacency matrix. From the results, we can conclude that some neighbors play a more important role than others in cancer subtype analyses of a particular sample based on the attention coefficient.

## 1. Introduction

Cancer is a complex and heterogeneous disease with hundreds of types and subtypes spanning across different organs and tissues, originating in various cell types [1,2]. For example, breast cancer is highly heterogeneous with different subtypes that lead to varying clinical outcomes, including prognosis, treatment response, and changes in recurrence and metastasis [3,4,5]. Hence, cancer subtype prediction and cancer patient stratification have been the subject of interest to clinicians and patients for many decades. Powered by high-throughput genomic technologies, the mRNA sequencing (RNA-seq) method is capable of measuring transcriptome-wide mRNA expressions and molecular activities in cancer cells [6,7]. Bulk RNA-seq data provide a view of an entire tissue sample’s average gene expression level instead of differentiating among cell types within the sample. Conversely, single-cell RNA-seq (scRNA-seq) provides opportunities to explore gene expression profiles at the single-cell level. These enable predicting the changes in expression level at a large scale to understand better the biological mechanism that leads to cancer.

The high-throughput RNA-seq datasets show quantitative measures of more than tens of thousands of mRNA isoforms for a cohort of hundreds or thousands of samples (e.g., patients, cells). However, due to the unavoidable sample heterogeneity or experimental noise in the data, extracting valuable biological information and discovering the underlying patterns from the data is becoming a serious challenge to computational biologists [8]. While hundreds of computational methods have been developed for cancer subtype prediction/identification [9,10] and patient stratification [11] using RNA-seq data [12,13], network analysis of sample similarities has largely been ignored in most methods. Recently, graph-based neural network (GNN) and network-based embedding models have shown remarkable success in learning network topological structures from large-scale biological data [14,15,16,17,18]. On another note, the self-attention mechanism has been extensively used in different applications, including bioinformatics [19,20,21]. This mechanism allows inputs to interact with each other and permits the model to utilize the most relevant parts of the inputs to improve the performance of the deep learning models. The self-attention mechanism was combined with the graph-structured data by Veličković et al. [22] in Graph Attention Networks (GAT). This GAT model calculates the representation of each node in the network by attending to its neighbors, and it uses multi-head attention to further increase the representation capability of the model [23]. It applies varied attentions to the neighbors; therefore, it finds the most important neighbors of a sample rather than giving all of them the same importance. This model has been successfully applied to various tasks, including text classification [24], node classification [25], social influence analysis [26], recommendation system [27], etc. The GAT model has also been applied to bioinformatics applications including drug-target interaction prediction [28], drug–microbe interaction prediction [29], gene essentiality prediction [30], scRNA-seq data dimensionality reduction [31], etc.

Inspired by the GAT model for capturing node dependencies in a wide range of domains, we propose the omicsGAT model and apply it to cancer samples with RNA-seq data. First, we test omicsGAT on The Cancer Genome Atlas (TCGA) breast invasive carcinoma (BRCA) data collections [32] and urothelial bladder carcinoma (BLCA) data collections [33] for cancer subtype prediction and cancer patient stratification, respectively (Section 2.1). Then, omicsGAT is applied on 2458 cells from six primary diffuse gliomas with K27M histone mutations (H3K27M) for cell clustering (Section 2.2). Next, we discuss and interpret the results based on the sample-by-sample attention matrix generated from the omicsGAT model (Section 3). Finally, we introduce the omicsGAT model in Section 4.

## 2. Results

We carry out experiments on TCGA RNA-seq datasets and scRNA-seq datasets to evaluate the performance of omicsGAT in this section. In the first part, we perform experiments with omicsGAT for cancer outcome prediction on TCGA breast cancer dataset and cancer patient stratification on TCGA bladder cancer dataset (Section 2.1). In the second part, omicsGAT is applied on scRNA-seq data of H3K27M-gliomas for single cell clustering analysis (Section 2.2).

### 2.1. Experiments on TCGA Cancer Patient Samples

#### 2.1.1. Datasets and Preprocessing

The proposed framework, omicsGAT, is tested on TCGA breast invasive carcinoma (BRCA) [32] and urothelial bladder carcinoma (BLCA) [33] datasets. The RNA-seq mRNA expression dataset of each cancer type was downloaded from UCSC Xena Hub [34]. log2(x+1) transformed mRNA expression is used in the analyses. The clinical information of the two cancer studies was downloaded from cBioPortal [35]. The BRCA dataset consists of 411 patient samples and 20,351 genes for each sample. Similarly, the BLCA dataset consists of 426 patient samples and 20,531 genes for each sample.

#### 2.1.2. omicsGAT Improved Overall Cancer Outcome Prediction

We design three tasks on TCGA BRCA mRNA expression data to evaluate the performance of the omicsGAT Classifier (Section 4.2) on cancer outcome prediction. There are 331 Estrogen Receptor positive (ER+) and 80 ER negative (ER−) samples, 284 Progesterone Receptor positive (PR+) and 127 PR negative (PR−) samples, and 65 Triple-negative (TN) and 346 non-TN samples in the dataset. The three tasks were to predict breast cancer patients’ ER, PR, and TN statuses. omicsGAT Classifier is compared with SVM, RF, DNN, GCN, and GraphSAGE. First, the dataset is divided into pre-train and test sets containing 80% and 20% of the total samples. Then, the pre-train set is divided into a training and validation set containing 80% and 20% samples of the pre-train set. The hyperparameters of the proposed model used in these two tasks are listed in Supplementary Table S1. They are selected through a grid search on the validation set. The same validation set is also applied to select the best model for DNN, GCN, and GraphSAGE. We run omicsGAT Classifier and baseline methods with the above-mentioned dataset splitting 50 times. The average AUROC scores for omicsGAT and baseline methods are reported in Table 1. As can be seen, our proposed model outperforms all the baselines for each of ER, PR, and TN status predictions. Moreover, the gain in AUROC caused by omicsGAT is significant in several cases. omicsGAT Classifier also offers a lower standard deviation, which signifies a more consistent and stable prediction compared to the baselines. The stability of our proposed model can be pertained to using several heads, which can secure information from different directions, and the model can effectively combine them by learning distinct attention parameters for each head.

**Figure 1:**
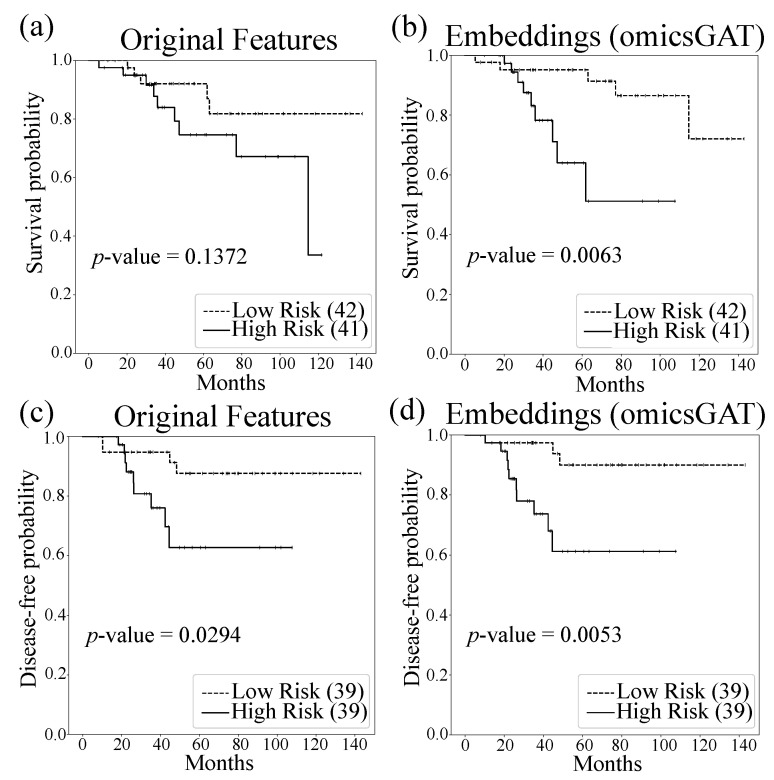
Survival and disease-free time predictions on breast cancer patients with original gene expression and the embeddings generated by omicsGAT. Kaplan–Meier plots for low (dashed line) and high (solid line) risk groups generated by (**a**) original gene expression and (**b**) omicsGAT learned embeddings for survival analysis; (**c**) original gene expression and (**d**) omicsGAT learned embeddings for disease-free analysis. The number in the parenthesis indicates the number of samples in the low- or high-risk group. The *p*-value is calculated by the log-rank test to compare the overall survival or disease-free probability of two groups of breast cancer patients.

To evaluate the performance of omicsGAT in greater depth, the patient’s overall survival time and disease-free time are predicted on the breast cancer dataset. The Cox proportional hazards model with elastic net penalty [36] evaluates the correlation between the patient’s overall survival time or disease-free time and genomic features (i.e., the original gene expression) or the omicsGAT learned embeddings. In total, 80% of the patient samples are applied to train the model and the performance is tested on 20% of the patient samples. The independent test set’s low and high risk groups were generated based on the prognostic index [37]. The survival and disease-free prediction are visualized by Kaplan–Meier plots and compared by the log-rank test. The Kaplan–Meier plots in Figure 1 illustrate the improved patient survival time and disease-free time prediction on breast cancer patients using the embeddings generated by omicsGAT compared to the original gene expression. The log-rank test *p*-values clearly demonstrate a strong additional prediction power of the learned embeddings beyond the gene expression.

#### 2.1.3. omicsGAT Improved Cancer Patient Stratification

To evaluate the generalization of our embedding mechanism, we employ omicsGAT Clustering (Section 4.3) to stratify bladder cancer (BLCA) patients. The dataset consists of five cancer subtypes, and our task is to cluster the patients into these five categories. Embeddings are generated following the first step of omicsGAT Clustering, i.e., an autoencoder. The hyperparameters stated in Table 2 are used to train the model for this task. First, the dimensions of the raw gene expression data are reduced using PCA implemented through *sklearn.decomposition.PCA* package. The top 400 PCA components are then used as input in the omicsGAT pipeline, and the generated embeddings are fed to the second step of omicsGAT Clustering, a hierarchical clustering model which would cluster the samples into different groups based on their embeddings.

**Figure 2:**
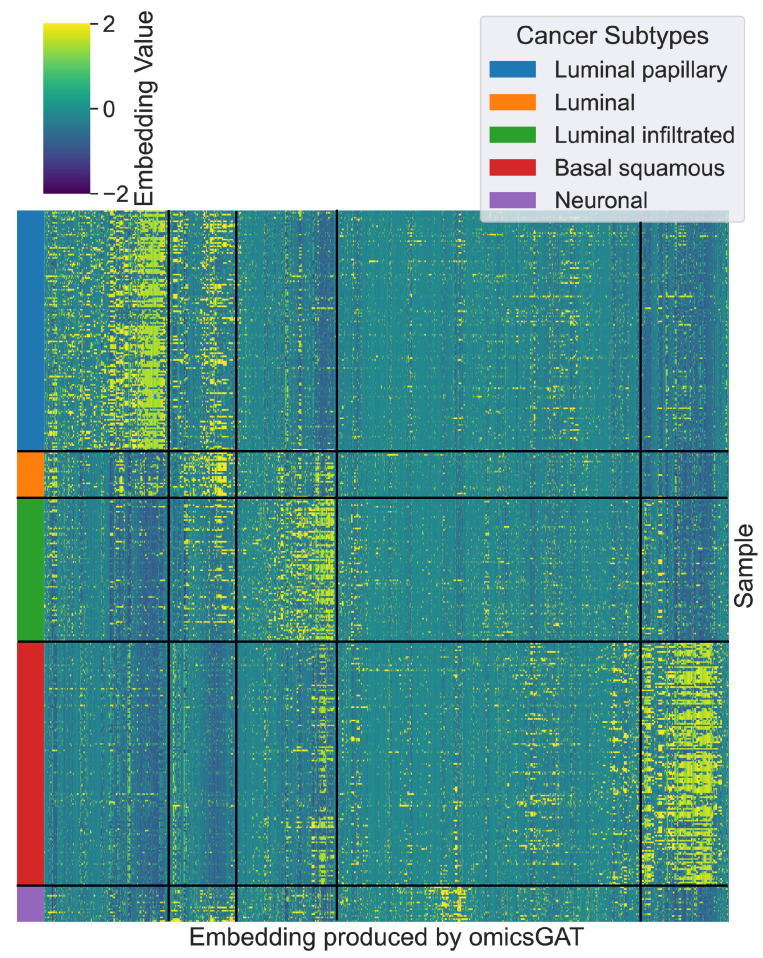
The embeddings generated by omicsGAT are clustered into the corresponding cancer subtypes.

Before clustering the samples into different groups, we first cluster the embeddings generated by omicsGAT, as illustrated in Figure 2. The patient samples are grouped according to their true cancer subtypes. The distinct pattern can be observed for the embeddings generated for a particular cancer subtype signifying the ability of omicsGAT to effectively integrate neighborhood information into the embedding for a better predictive signature.

Next, we compare the performance of omicsGAT Clustering with the five baselines (Section 4.4.2) for clustering patient samples into cancer subtypes. Hierarchical and k-means clustering algorithms are applied to the raw gene expression, their 400 PCA components, and the adjacency matrix. NMI and ARI scores are computed based on the assigned clusters. The same procedure is followed for the embeddings generated by omicsGAT and the trained encoders of DNN-based and GCN-based autoencoders. The results are reported in Table 3. It can be observed that both NMI and ARI scores are highest for omicsGAT Clustering, followed by that of the adjacency matrix and GCN-based autoencoder. These three methods consider the relation between the samples, which helps the downstream clustering models to form better clusters. On the other hand, the scores for the raw gene expression, PCA components, and the embeddings generated by the DNN-based autoencoder are lower, which can be attributed to the absence of sample similarity information. omicsGAT uses the information from the neighbors more effectively by assigning different attention coefficients to the neighbors of a sample, thereby capturing the hidden relations between samples in the embeddings. This influx of information caused by the attention mechanism in embedding generation enables omicsGAT Clustering to outperform all baselines by a considerable margin.

**Figure 3:**
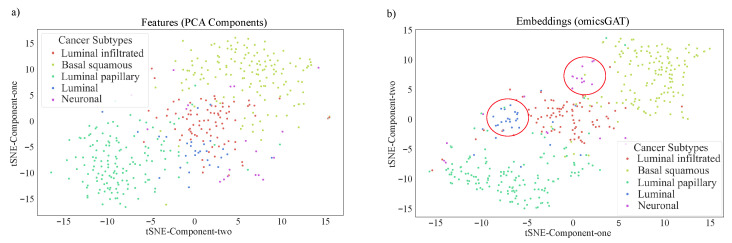
tSNE plots of the (**a**) PCA components generated from the BLCA data and (**b**) omicsGAT-generated embeddings for bladder cancer patients stratification.

To visualize the clustering performance, tSNE plots (Python *seaborn* [38] package) are created on the PCA components and the embeddings generated by omicsGAT, in Figure 3a and Figure 3b, respectively. Figure 3a illustrates that PCA components cannot properly separate the five clusters. Although there is some separation among the patient samples in ‘Basal squamous’, ‘Luminal papillary’, and ‘Luminal infiltrated’ subtypes, the samples in ‘Luminal’ and ‘Neuronal’ subtypes are randomly scattered on the plot. On the other hand, Figure 3b shows that omicsGAT Clustering can effectively separate all five clusters, revealing the meaningful neighborhood information contained within the embeddings. Moreover, ‘Luminal’ and ‘Neuronal’ are the subtypes with the smallest number of samples, which means our proposed method particularly excels at clustering rare subtypes.

### 2.2. Experimentation on Single-Cell RNA-seq Data

Single-cell RNA-seq (scRNA-seq) data reveals heterogeneity at the cell level and offers a more significant number of samples (i.e., cells) compared to bulk RNA-seq data (e.g., number of patient samples). We apply omicsGAT Clustering on scRNA-seq data and cluster cells to evaluate the generalization of our proposed model.

#### 2.2.1. Dataset and Preprocessing

scRNA-seq data from six primary H3K27M-gliomas (H3 lysine27-to-methionine mutations) are used in the following experiment. This type of gliomas (malignant tumors) primarily arises in the midline of the central nervous system of young children [39]. Early detection of tumors may improve disease prognosis; hence, stratifying the tumor cells into the correct gliomas could be very helpful for clinicians. Gene expression and label information of 2458 cells is used for this experiment. The dataset was downloaded from the Single Cell Portal [39], which consists of the cells generated from six different gliomas: BCH836, BCH869, BCH1126, MUV1, MUV5, and MUV10. log2(x+1) transformed TPM (Transcripts-per-million) value is used in the analysis.

#### 2.2.2. Single Cell Clustering

**Figure 4:**
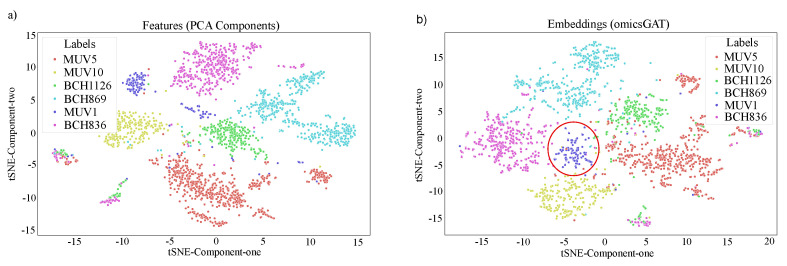
tSNE plots of the (**a**) PCA components generated from the ‘H3K27M-gliomas’ scRNA-seq data and (**b**) omicsGAT-generated embeddings for cell clustering.

The same omicsGAT Clustering method (Section 4.3) is followed to cluster the cells with scRNA-seq data. The top 200 PCA components are selected as the input of the omicsGAT Clustering to generate embeddings. The omicsGAT’s hyperparameters for this experiment are listed in Supplementary Table S2. The autoencoder is trained following the same steps as explained in Section 2.1.3. Embeddings generated by the autoencoder are then fed into the hierarchical clustering model. Hierarchical and k-means clustering methods on raw gene expression, PCA components, and the embeddings generated by the DNN-based and GCN-based autoencoders are considered as the baselines, along with hierarchical clustering on the adjacency matrix. Moreover, SC3s [40], a consensus clustering method for scRNA-seq data analysis, is also considered as a baseline for better evaluation of omicsGAT’s performance on single cell clustering. As reported in Table 4, omicsGAT Clustering outperforms all the baselines, meaning the cluster assignments resulting from the omicsGAT-generated embeddings are more similar to the true label information. This result is corroborated by the tSNE plots in Figure 4a,b, which are drawn on the PCA components and the embeddings generated by omicsGAT, respectively. The tSNE plot for omicsGAT Clustering shows more separation among the clusters as compared to the PCA components. Specifically, for the ‘MUV1’ group, our model forms a single cluster containing all the cells belonging to that type (red circle in Figure 4b), whereas the tSNE plot using PCA components shows two different clusters for the cells in ‘MUV1’. Based on the results, we can conclude that in the case of scRNA-seq data analysis, omicsGAT Clustering can take advantage of the detailed cellular level information and uses the attention mechanism on the cell-cell similarity network to cluster the samples better.

## 3. Discussion

omicsGAT can successfully integrate the structural information within gene expression data into sample embeddings, enabling better classification and clustering performance compared to the original dataset. The self-attention mechanism in omicsGAT contributes to the stronger predictive ability of the embeddings. A binary adjacency matrix is applied to define neighborhoods in omicsGAT that includes self-connections to ensure that the information of a sample itself is also considered in the embedding. The performance is reduced when we run the same tasks with just the adjacency matrix. The adjacency matrix is calculated using correlation only, which keeps track of the pairwise linear relations between samples. However, using the attention mechanism, omicsGAT can capture complex nonlinear relations by accounting for the importance of neighboring samples on the classification or clustering of a target sample. The captured relations among samples are represented in the generated embeddings, enabling the model to perform better on classification and clustering tasks.

**Figure 5:**
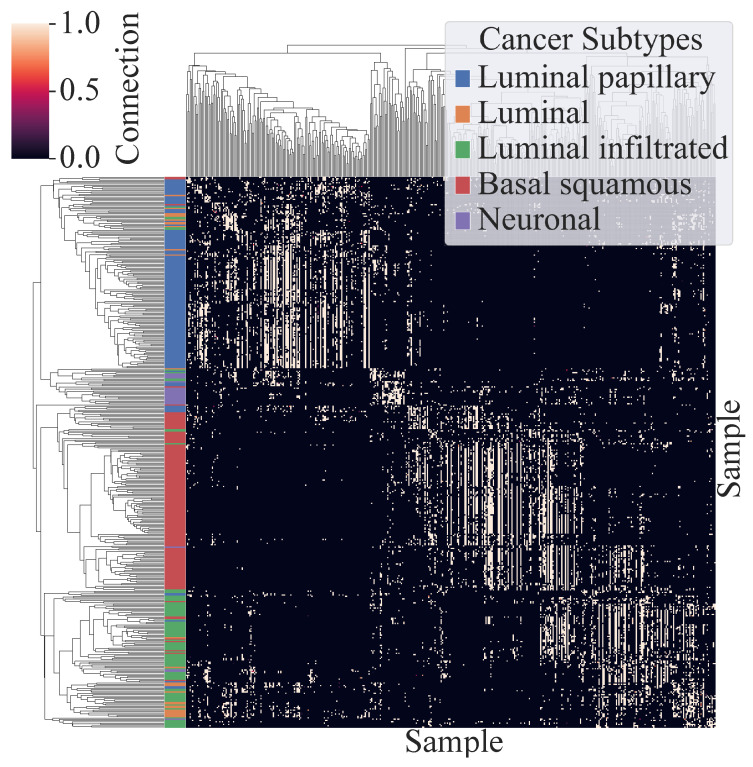
Clustermap of the Attention Matrix generated from the trained omicsGAT model on BLCA data.

In order to verify the effect of the multi-head attention mechanism, a sample×sample attention matrix is constructed by extracting the attention coefficients from a trained omicsGAT model following the method used by Ullah and Ben-Hur [41]. For a target sample, each of the *h* heads assigns different attention coefficients to its neighbors, and only the highest among the *h* attention coefficients is considered for each neighbor to represent its relation with the target sample. The same procedure is repeated to generate the full attention matrix. This process is applied to build the attention matrix for both BLCA and cell clustering tasks described in Section 2.1.3 and Section 2.2.2, respectively. This attention matrix reveals the importance of combining the attention mechanism with the network information received through the adjacency matrix. As seen in Table 5, clustering on the attention matrix outperforms the clustering on the adjacency matrix for both datasets. Moreover, the clustermap of the attention matrix obtained from the trained model on BLCA data, illustrated in Figure 5, shows a distinct pattern of the cancer subtypes specifically for ‘Luminal papillary’ and ‘Basal squamous’ subtypes. From these results, we can conclude that some neighbors play a more important role than others in classification or clustering of a sample, and omicsGAT can effectively inject this information into the model along with the graph structure to generate more meaningful embeddings for better downstream analyses. An important aspect of omicsGAT is the use of multiple heads. The learnable weight parameters (W and A) of each head are initialized separately using the *xavier normal* library function of *Pytorch* [42].

For the clustering tasks, the NMI and ARI scores of the baselines are relatively low with hierarchical clustering, which can be observed in Table 3 and Table 4. Therefore, we also apply k-means clustering on them in order to compare them with omicsGAT. Since the performance of k-means clustering depends on the initialization of the cluster-centers, clustering is conducted ten times, and the mean scores along with standard deviations are reported in the tables.

To evaluate the clustering performance of omicsGAT in greater depth, it is also applied on the latest SARS-CoV-2 antibodies scRNA-seq data [43], consisting of 6050 cells. The hyperparameters used for this experiment are stated in Supplementary Table S3. The same baselines as mentioned in Section 2.2.2 are used for comparison in this experiment. The NMI and ARI scores computed on the hierarchically clustered omicsGAT-generated embeddings along with that of the other baselines are reported in Supplementary Table S4. omicsGAT surpasses the other models in this scenario as well. This result also generalizes the applicability of omicsGAT to the domain of other diseases. The respective tSNE plots of the PCA components and omicsGAT-generated embeddings for this dataset are provided in Supplementary Figure S1.

## 4. Methods

In this section, we first introduce our proposed framework, omicsGAT, which generates embeddings from gene expression data to be used in downstream classification and clustering. We extend the GAT model [22] to better fit our tasks of disease outcome prediction and subtype stratification. Then, we discuss the baseline models used to compare and validate the performance of omicsGAT, followed by the details of evaluation metrics used in this study.

### 4.1. Graph Attention Network

The omicsGAT model architecture builds on the concept of the self-attention mechanism. In omicsGAT, embedding is generated from the gene expression data, assuming that the samples (i.e., patients or cells) with similar features (gene expressions) are expected to have similar disease outcomes or cell types, and therefore, are connected to each other. Hence, network information is injected into the model using the adjacency matrix to consider these connections. However, all connected neighbors of a target sample should not get equal attention in generating the embedding for that sample. A particular neighbor of a target sample can contribute more to its subsequent prediction or clustering, which similarity metrics cannot accurately apprehend. Therefore, to capture the importance of each neighbor on a sample, the omicsGAT model automatically assigns different attentions to the neighbors of that sample for a singular head while generating the embedding. Moreover, to consider the impact of different types of information secured from the neighbors and stabilize the learning process, the above procedure is repeated multiple times in parallel employing several heads (independent attention mechanisms) in a multi-head framework.

The mathematical notations used to explain omicsGAT are summarized in Table 6. Let *n* be the number of samples (e.g., patients, cells) and *m* be the number of features (e.g., genes) representing each sample. The input feature matrix is given by $X=[x_{1},x_{2},...,x_{n}]$, where $x\in R^{1\times m}$ represents a sample vector. Let A be the n×n adjacency matrix (includes self-connections) built based on the pairwise correlation between the samples. Suppose that the set of neighbors for a sample $x_{i}$ is denoted by $N_{i}$. Depending on the number of neighbors $|N_{i}|$ to be kept for a sample, the connections with high correlation scores are kept (assigned a value of 1), and the others are discarded (assigned a value of 0). The adjacency matrix is binarized, as it will be used to mask the attention coefficients in later part of the model. Self-connections are applied to integrate the information from the samples themselves in their embeddings. While generating the embedding of sample $x_{i}$, the attention given to it from its neighbor $x_{j}$ for a single head can be calculated as:

(1) $$c_{ij}=a^{T}[Wx_{i}||Wx_{j}]$$

where $W\in R^{p\times m}$ and $a\in R^{2p\times 1}$ are learnable weight parameters of a single head, which are shared across all the samples and *p* is the embedding size, and || and $.^{T}$ symbols denote the concatenation and transposition operations of the matrices, respectively. The calculated attention values are passed through a *LeakyReLU* activation function. Then, the structural information of the network is introduced by masking the attention values using the adjacency matrix. Only the attention values where a connection is present between the nodes (samples) in the adjacency matrix A are kept, and all the remaining values are made zero. After that, the attention coefficient for a neighbor $x_{j}$ is calculated using a *Softmax* function, which follows the equation below:

(2) $$\alpha_{ij}=\frac{\exp(\mathrm{LeakyReLU}(a^{T}[Wx_{i}||Wx_{j}]))}{\sum_{r\in N_{i}}\exp(\mathrm{LeakyReLU}(a^{T}[Wx_{i}||Wx_{r}]))}.$$

The attention coefficients calculated for all of the neighbors of $x_{i}$ using Equation (2) are leveraged to calculate its final embedding for a single head:

(3) $${x^{\prime }}_{i}=\sigma \left(\sum_{j\in N_{i}}\alpha_{ij}Wx_{j}\right)$$

where σ is a non-linear activation function. Note that the sample $x_{i}$ is also included in its neighbors since self-connections are used in the adjacency matrix.

**Figure 6:**
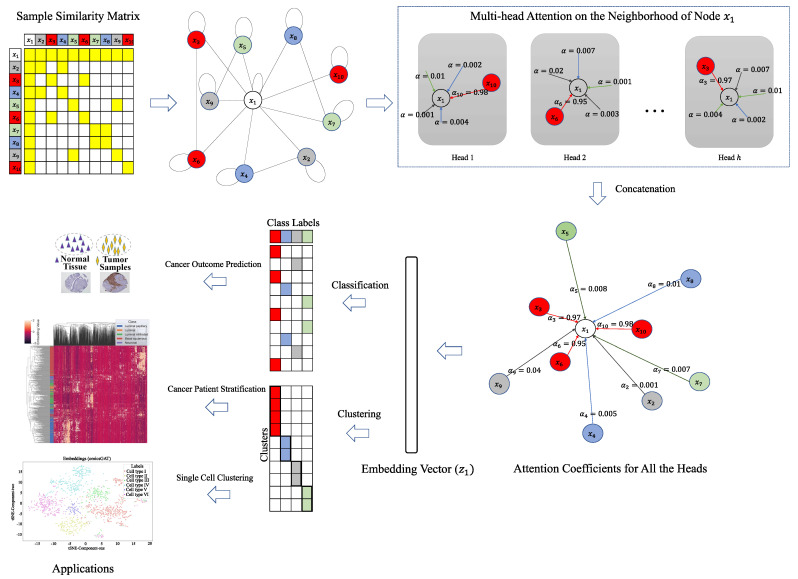
Workflow of omicsGAT. For a sample $x_{1}$, based on the input feature matrix and adjacency matrix, each head calculates the attention given to $x_{1}$ from its neighbors separately. The embeddings produced by all heads are concatenated together to generate the final embedding $z_{1}$ for $x_{1}$, which is then used for classification or clustering of $x_{1}$.

In a multi-head attention network, each head has a separate attention mechanism with its own weight matrix W and attention vector a. Outputs generated by all the heads for one particular sample are concatenated to generate the final embedding vector of that sample. This is done to stabilize the learning process while generating the embedding. This is similar to the mechanism used by Vaswani et al. [19] in self-attention. Hence, the output embedding from the first part of our model for $x_{i}$ is given by:

(4) $$z_{i}=|{|}_{k=1}^{h}\sigma \left(\sum_{j\in N_{i}}\alpha_{ij}^{k}W^{k}x_{j}\right)$$

where *h* is the number of heads. The output projected in the embedding space is represented by $Z\in R^{n\times ph}$, and embedding for one sample is $z\in R^{1\times ph}$. The generated embeddings are then used in separate models for classification and clustering tasks. The overall framework of our proposed pipeline is illustrated in Figure 6.

### 4.2. omicsGAT Classifier

omicsGAT Classifier is a unified model that passes the embedding Z generated from the first part of our pipeline described in Section 4.1 through a fully connected (FC) layer followed by a *Softmax* function. Let the number of classes for the classification task be *c*. The FC layer transforms $Z\in R^{n\times ph}$ into $Y_{cls}\in R^{n\times c}$, where $Y_{cls}=\left[y_{cls_{1}},y_{cls_{2}},...,y_{cls_{n}}\right]$ represents the classification outcomes. It can be formulated as:

(5) $$Z_{cls}=\mathrm{Softmax}\left(W_{cls}Z_{in}+b_{cls}\right)$$

where $Z_{cls}$ and $Z_{in}$ are the output and input matrices, $W_{cls}$ is the learnable weight, and $b_{cls}$ is the bias vector of the FC layer.

Let the ground truth labels for *n* samples be $Y=[y_{in_{1}},y_{in_{2}},...,y_{in_{n}}]$. In order to calculate the overall loss of the model, the Negative Log Likelihood (NLL) loss function is applied, formulated as follows:

(6) $$L_{cls}=-\sum_{i=1}^{n}\log\left(\mathrm{Likelihood}\left(y_{cls_{i}}|y_{in_{i}}\right)\right).$$

where $L_{cls}$ is minimized to train the unified omicsGAT Classifier framework.

### 4.3. omicsGAT Clustering

For clustering, we propose a two-step omicsGAT Clustering framework. The first step is an autoencoder that generates the gene expression embedding in an unsupervised approach, and the second step is a hierarchical clustering model. omicsGAT described in Section 4.1 serves as the encoder in the autoencoder architecture whereas a four layers fully connected neural network is constructed as the decoder. The output $Z\in R^{n\times ph}$ from the omicsGAT encoder is fed into the first layer of the decoder. The output of the consecutive FC layers are $Z_{clr_{1}}\in R^{n\times \frac{ph}{2}}$, $Z_{clr_{2}}\in R^{n\times \frac{m}{4}}$, $Z_{clr_{3}}\in R^{n\times \frac{m}{2}}$, and $Y_{clr}\in R^{n\times m}$, respectively. Each layer can be formulated as:

(7) $$Z_{clr}=\sigma \left(W_{clr}Z_{in}+b_{clr}\right)$$

where $Z_{clr}$ and $Z_{in}$ are the output and input matrices, $W_{clr}$ is the learnable weight, and $b_{clr}$ is the bias vector of a particular layer of the decoder. For the first three layers, σ denotes the activation function *ReLU*, and no activation function is used in the final layer.

The output, projected back to the input feature space by the decoder, is given by $Y_{clr}=\left[y_{clr_{1}},y_{clr_{2}},...,y_{clr_{n}}\right]$. The mean squared error (MSE) is employed to calculate the reconstruction loss as follows:

(8) $$L_{clr}=\sum_{i=1}^{n}{\left(x_{i}-y_{clr_{i}}\right)}^{2}.$$

where $L_{clr}$ is minimized to train the autoencoder, and an embedding is generated as output from the trained encoder. The embedding is then fed into the second step of omicsGAT Clustering, a hierarchical clustering model implemented using the *scikit-learn* package [44]. It stratifies the input samples into a defined number of clusters by assigning each sample to a group based on the similarity of the generated embedding with that of the other samples in the group.

### 4.4. Baseline Models Used for Comparison

#### 4.4.1. Baselines for Classification Tasks

Support Vector Machine (SVM), Random Forest (RF), Deep Neural Network (DNN), Graph Convolutional Network (GCN) [45], and GraphSAGE [46] are used as baselines to evaluate and compare the performance of the omicsGAT Classifier. The baselines are built using several Python open-source library packages, including *Scikit-learn* [44], *PyTorch* [42], and *PyTorch Geometric* [47].

SVM and RF are two of the most widely used machine learning models. In this study, ‘rbf’ kernel is applied for SVM. Hyperparameters for RF, including the number of trees, split criterion, maximum depth of the tree, and maximum number of features considered for split, are also tuned. The Deep Neural Network model consists of three fully connected linear layers, with the first two of them followed by the *ReLU* activation function. For better evaluation of our model by comparing it to similar graph-based deep learning models, we follow the GCN proposed by Kipf and Welling [45] and GraphSAGE (SAmple and aggreGatE) proposed by Hamilton et al. [46]. Both models are composed of a GCN/GraphSAGE layer followed by two FC layers. The correlation-based adjacency matrix A is used as neighborhood information in these models. The hyperparameters for all of these models are tuned on the validation set using grid search.

#### 4.4.2. Baselines for Clustering Tasks

The performance of embeddings generated by omicsGAT for the downstream clustering task is evaluated against embeddings generated by a DNN-based autoencoder and a GCN-based autoencoder. The encoder part in the autoencoders consists of the respective model, and the decoder part comprises three FC layers. Hierarchical or k-means clustering is employed on the embeddings generated by the trained encoders of the baselines. Furthermore, to get a better understanding of the improvements made by omicsGAT, clustering of the raw features (gene expression), their PCA components, and adjacency matrix are compared with omicsGAT-generated embeddings. In addition to the aforementioned models, an efficient clustering method targeted for scRNA-seq data named SC3s [40] is used as a baseline for the single-cell clustering task.

### 4.5. Evaluation Metrics

In this section, we define three evaluation metrics used in this study implemented using the *scikit-learn* library of Python. The Area Under the Receiver Operating Characteristic Curve (AUROC) is used for the comparison of the classification models. It is defined as the area under the curve plotted using True Positive Rate (*precision*) along the y-axis and False Positive Rate (1-*specificity*) along the x-axis. The Normalized Mutual Information (NMI) and Adjusted Rand Index (ARI) are applied to evaluate the clustering methods, both ranging from 0 to 1, where 1 means perfect clustering and 0 means totally random.

## 5. Conclusions

Powered by high-throughput genomic technologies, the RNA-seq method is capable of measuring transcriptome-wide mRNA expressions and molecular activities in cancer cells. Hundreds of computational methods have been developed for cancer outcome prediction, patient stratification, and cancer cell clustering. Some of these methods consider sample-sample similarities in the analysis, and some of them do not. These sample similarity-based methods cannot distinguish the importance of the neighbors for a particular sample in the downstream prediction or clustering tasks. Therefore, we introduce omicsGAT in this study, which leverages a self-attention mechanism consisting of multiple heads to assign proper attention weights to the neighbors of a sample in the network. Experiments on cancer subtype analyses show the superior performance of the model in every aspect compared to the baseline methods. We also show the generalization of omicsGAT’s performance on both bulk RNA-seq and scRNA-seq data. As a future objective, we would like to extend omicsGAT to include metapath selection, which would consider the best paths in a network to perform a certain task on a particular sample.

## Figures and Tables

**Table 1:** The classification performance on TCGA breast cancer (BRCA) dataset. The mean AUROC scores and standard deviation (SD) of classifying patients in breast cancer subtypes are reported. * Denotes the difference between the results of omicsGAT and baseline method to be statistically significant (*p*-value < 0.001).

Cancer Subtype	Method	AUC Score	SD
ER	SVM	0.9089 *	0.0414
	Random Forest	0.9177 *	0.0408
	DNN	0.9498	0.0337
	GCN	0.9581	0.0289
	GraphSAGE	0.9493	0.0288
	**omicsGAT**	**0.9636**	**0.0215**
PR	SVM	0.8199 *	0.0456
	Random Forest	0.8475 *	0.0476
	DNN	0.8741 *	0.0405
	GCN	0.8847 *	0.0441
	GraphSAGE	0.8875	0.0450
	**omicsGAT**	**0.9065**	**0.0439**
TN	SVM	0.8905 *	0.0614
	Random Forest	0.8515 *	0.0609
	DNN	0.9419 *	0.0400
	GCN	0.9492	0.0269
	GraphSAGE	0.9527	0.0243
	**omicsGAT**	**0.9611**	**0.0219**

**Table 2:** Hyperparameter selection for omicsGAT Clustering. The bolded values are used as defaults.

Hyperparameter	Selection Set
No. of PCA components (features) selected	[50,100,200,400]
Embedding size of a head	[4,8,16,32,64]
No. of heads	[4,8,16,32,64]
Network density of adjacency matrix	[0.02,0.04,0.1,0.2]
No. of FC layers	[2,3,4]

**Table 3:** The clustering performance on TCGA bladder cancer (BLCA) dataset. The NMI and ARI scores of omicsGAT Clustering and baseline methods are reported in the table. Hierarchical clustering was computed with ‘Manhattan’ distance and ‘Average’ linkage. Mean NMI and ARI scores with standard deviation (SD) are reported for k-means clustering (run 10 times).

Input Data (Clustering Method)	NMI	NMI SD	ARI	ARI SD
gene expression (hierarchical)	0.0515	-	0.0153	-
gene expression (k-means)	0.4944	0.0171	0.4468	0.0548
PCA components (hierarchical)	0.1222	-	0.0353	-
PCA components (k-means)	0.4883	0.0176	0.4338	0.0388
DNN-based autoencoder (hierarchical)	0.1471	-	0.0380	-
DNN-based autoencoder (k-means)	0.4544	0.0164	0.4879	0.0301
GCN-based autoencoder (hierarchical)	0.1697	-	0.1645	-
GCN-based autoencoder (k-means)	0.5146	0.0164	0.4879	0.0025
adjacency matrix (hierarchical)	0.5448	-	0.5505	-
**omicsGAT embeddings** (hierarchical)	**0.6147**	-	**0.6698**	-

**Table 4:** The clustering performance on H3K27M-gliomas scRNA-seq data. The NMI and ARI scores of omicsGAT Clustering and baseline methods are reported in the table. Hierarchical clustering was computed with ‘Cosine’ distance and ‘Average’ linkage. Mean NMI and ARI scores with standard deviation (SD) are reported for k-means clustering (run 10 times).

Matrix Type (Clustering Type)	NMI	NMI SD	ARI	ARI SD
gene expression (hierarchical)	0.0055	-	0.0010	-
gene expression (k-means)	0.5052	0.0176	0.4410	0.0145
PCA components (hierarchical)	0.6146	-	0.5339	-
PCA components (k-means)	0.5010	0.0016	0.4640	0.0013
DNN-based autoencoder (hierarchical)	0.6304	-	0.5687	-
DNN-based autoencoder (k-means)	0.6086	0.0226	0.5296	0.0384
GCN-based autoencoder (hierarchical)	0.5366	-	0.4133	-
GCN-based autoencoder (k-means)	0.5110	0.0431	0.3610	0.0568
SC3s	0.6077	-	0.5457	-
adjacency matrix (hierarchical)	0.5757	-	0.3982	-
**omicsGAT embeddings** (hierarchical)	**0.6584**	-	**0.6366**	-

**Table 5:** NMI and ARI scores of the Hierarchical Clustering applied on attention and adjacency matrices.

Dataset	Input Matrix	NMI	ARI
BLCA	adjacency matrix	0.5448	0.5505
	attention matrix	0.5743	0.6373
H3K27M	adjacency matrix	0.5757	0.3982
	attention matrix	0.5788	0.4821

**Table 6:** Mathematical notations for omicsGAT.

Name	Definition
*n*	number of samples (i.e., patients or cells)
*m*	number of features (i.e., genes)
*p*	embedding size for a single head
*h*	number of heads
$X\in R^{n\times m}$	input feature matrix
$A\in R^{n\times n}$	correlation-based adjacency matrix of samples
$W\in R^{m\times p}$	weight matrix of a single head
$a\in R^{2p\times 1}$	attention weight matrix of a single head
$\alpha \in R^{n\times n}$	attention coefficients of a single head
$Z\in R^{n\times ph}$	embedding matrix learned from the model

## Data Availability

Source code is freely available at: https://github.com/compbiolabucf/omicsGAT (accessed on 3 September 2022).

## Supplementary Materials

The following supporting information can be downloaded at: https://www.mdpi.com/article/10.3390/ijms231810220/s1.
