# Supplementary material for: omicsGAT: Graph Attention Network for Cancer Subtype Analyses
Source: Int J Mol Sci. 2022 Sep 6;23(18):10220. doi: 10.3390/ijms231810220 (PMC9499656; doi:10.3390/ijms231810220)
Supplement: Supplementary file 1 [file ijms-23-10220-s001.zip › ijms-1848649-supplementary.pdf]

Supplementary Materials for "omicsGAT: Graph Attention Network for Cancer Subtype Analyses"

1 Tables

Table S1: Sets of hyperparameters in omicsGAT for cancer outcome prediction

Hyperparameter	Selection Set
No. of features selected using correlation	$[128, 256, 512, 1024]$
Embedding size of a head	$[4, 8, 16, 32, 64]$
No. of heads	$[4, 8, 16, 32, 64]$
No. of neighbors of a sample in the adjacency matrix	$[5, 10, 20, 40]$

Table S2: omicsGAT's hyperparameter selection for 'H3K27M-gliomas' single-cell clustering. The bold hyperparameters are selected for the clustering task based on the least reconstruction loss of the decoder.

Hyperparameter	Selection Set
No. of PCA components (features) selected	$[50, 100, 150, \mathbf{200}, 400]$
Embedding size of a head	$[4, \mathbf{8}, 12, 16, 32, 64]$
No. of heads	$[4, \mathbf{8}, 12, 16, 32, 64]$
No. of neighbors of a node in adjacency matrix	$[5, \mathbf{10}, 20, 40, 70]$
No. of FC layers	$[2, \mathbf{3}, 4]$

Table S3: omicsGAT's hyperparameter selection for 'SARS-CoV-2 Anbodies' single-cell clustering. The bold hyperparameters are selected for the clustering task based on the least reconstruction loss of the decoder.

Hyperparameter	Selection Set
No. of PCA components (features) selected	$[50, 100, \mathbf{150}, 200, 400]$
Embedding size of a head	$[4, 8, \mathbf{12}, 16, 32, 64]$
No. of heads	$[4, 8, \mathbf{12}, 16, 32, 64]$
No. of neighbors of a node in adjacency matrix	$[5, 10, 20, 40, \mathbf{70}]$
No. of FC layers	$[2, \mathbf{3}, 4]$

Table S4: The clustering performance on SARS-CoV-2 antibodies scRNA-seq data. The NMI and ARI scores of omicsGAT Clustering and baseline methods are reported in the table. Hierarchical clustering was computed with 'Euclidean' distance and 'Ward' linkage. Mean NMI and ARI scores with standard deviation are reported for k-means clustering (run 10 times).

Matrix Type (Clustering Type)	NMI	NMI SD	ARI	ARI SD
gene expression (hierarchical)	0.5558	-	0.4655	-
gene expression (k-means)	0.5923	0.0419	0.5012	0.0626
PCA components (hierarchical)	0.5920	-	0.5167	-
PCA components (k-means)	0.6409	0.0035	0.5524	0.0032
DNN-based autoencoder (hierarchical)	0.5240	-	0.4147	-
DNN-based autoencoder (k-means)	0.6029	0.0002	0.5005	0.0002
GCN-based autoencoder (hierarchical)	0.2870	-	0.1606	-
GCN-based autoencoder (k-means)	0.2979	0.0002	0.1726	0.0002
SC3s	0.6164	-	0.5223	-
adjacency matrix (hierarchical)	0.3696	-	0.2739	-
**omicsGAT embeddings** (hierarchical)	**0.6677**	-	**0.6730**	-

2 Figures

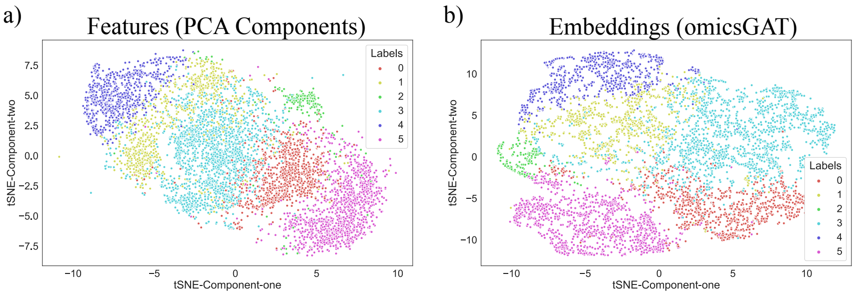

Figure S1: **tSNE plots** of the (a) PCA components generated from the 'SARS-CoV-2 antibodies' scRNA-seq data and (b) omicsGAT generated embeddings for cell clustering.
